# Physical activity in subjects with multiple sclerosis with focus on gender differences: a survey

**DOI:** 10.1186/1471-2377-14-47

**Published:** 2014-03-10

**Authors:** Elisabeth Anens, Margareta Emtner, Lena Zetterberg, Karin Hellström

**Affiliations:** 1 Department of Neuroscience, Section for Physiotherapy, Uppsala University, Uppsala, Sweden; 2 Department of Medical Sciences, Respiratory Medicine and Allergology, Uppsala University, Uppsala, Sweden

**Keywords:** Multiple sclerosis, Gender, Physical activity, Self-efficacy, Fatigue, Social support

## Abstract

**Background:**

There is increasing research that examines gender-issues in multiple sclerosis (MS), but little focus has been placed on gender-issues regarding physical activity. The aim of the present study was to describe levels of physical activity, self-efficacy for physical activity, fall-related self-efficacy, social support for physical activity, fatigue levels and the impact of MS on daily life, in addition to investigating gender differences.

**Methods:**

The sample for this cross-sectional cohort study consisted of 287 (84 men; 29.3%) adults with MS recruited from the Swedish Multiple Sclerosis Registry. A questionnaire was sent to the subjects consisting of the self-administrated measurements: Physical Activity Disability Survey – Revised, Exercise Self-Efficacy Scale, Falls- Efficacy Scale (Swedish version), Social Influences on Physical Activity, Fatigue Severity Scale and Multiple Sclerosis Impact Scale. Response rate was 58.2%.

**Results:**

Men were less physically active, had lower self-efficacy for physical activity and lower fall-related self-efficacy than women. This was explained by men being more physically affected by the disease. Men also received less social support for physical activity from family members. The level of fatigue and psychological consequences of the disease were similar between the genders in the total sample, but subgroups of women with moderate MS and relapsing remitting MS experienced more fatigue than men.

**Conclusions:**

Men were less physically active, probably a result of being more physically affected by the disease. Men being more physically affected explained most of the gender differences found in this study. However, the number of men in the subgroup analyses was small and more research is needed. A gender perspective should be considered in strategies for promoting physical activity in subjects with MS, e.g. men may need more support to be physically active.

## Background

The incidence and prevalence of multiple sclerosis (MS) has increased over the past decades, particularly among women [1]. The prevalence in Sweden is currently 188.9/100 000 [2] and about 75% debut between 20–40 years of age [3]. MS is a heterogeneous progressive neurological disorder with consequences such as muscle weakness, loss of balance, limited sensation, fatigue, and spasticity that can limit a person’s ability to be physically active [3]. MS is a demyelinating disease of the central nervous system, and the demyelinated areas are characterized by inflammation [4]. Physical inactivity in subjects with MS can lead to deterioration in physical function and increased risk of secondary illness such as cardiovascular co-morbidities [5,6]. Physical activity can be defined as all bodily movement that derives from the contraction of the skeletal muscles and results in increased energy expenditure [7]. Subjects with MS are less physically active than the general population [8-10], and individuals with more severe disease are less physically active compared to those with a milder disease [11]. Persons with MS have lower accelerometer activity and step counts, and less time spent in moderate-to vigorous physical activity than matched controls [9]. Cardiorespiratory exercise capacity was up to 30% lower in persons with MS than in healthy controls [12]. There is strong evidence that physical activity has positive effects on physical and mental health and quality of life [13-15]. Positive effects of physical activity have also been demonstrated in people with MS e.g. muscle strength, aerobic capacity, mobility, fatigue level, walking, and quality of life [16-20].

An important facilitator for physical activity, self-efficacy for physical activity [21,22], has also been observed in subjects with MS [23-25] but not in all samples [11,26]. Self-efficacy is defined as the conviction that one can successfully execute the behavior required to produce a desired outcome and is a central concept in Social Cognitive Theory [27,28]. Another type of self-efficacy is fall-related self-efficacy, defined as the degree of efficacy (i.e. self-confidence) to avoid a fall [29]. Fall-related self-efficacy is relevant in subjects with MS since falling and fear of falling is often reported [30-32]. Fatigue has also been found to be a barrier to physical activity in some studies [23,25] but not in all [11,33]. On the other hand, social support for physical activity can be a facilitator for physical activity in subjects with MS [34], however, contradictory results have been reported [11]. Studies regarding facilitators and barriers to physical activity in subjects with MS are still few and not consistent.

In the general population it has been shown that men have a higher physical activity level than women [35]. Gender differences regarding factors associated with physical activity have also been shown, such as personal barriers that have been found to be more important to women than men [36-38]. A meta analysis [8] of physical activity in individuals with MS showed combined samples of women and men to be significantly less physically active compared to samples of women alone, indicating that men are less physically active. Jobin et al. [39] reviewed the literature and summarized gender-related issues in MS, showing that a benign course of the disease is more often associated with the female gender, and the progression is worse for men than for women when all disease courses are included. On the other hand men and women experience disabling fatigue with the same frequency, whereas studies investigating gender in social support in general show varying results [39]. Little research focus has been placed on gender in MS with regard to physical activity, despite the gender differences found in other populations.

### Aims

The aim of the study was to examine physical activity, severity of disease, fatigue levels, self-efficacy for physical activity, fall-related self-efficacy and social support for physical activity, in a sample of subjects with MS, in addition to study gender differences.

## Methods

The study has been performed as a cross-sectional cohort survey with a descriptive and comparative design.

### Study cohort

Individuals between 18–80 years with a diagnosis of MS were recruited from the Swedish Multiple Sclerosis Registry (http://www.msreg.net). The register specify four types of MS; relapsing remitting, secondary progressive, primary progressive or progressive relapsing. All registered subjects living in the county of Uppsala were invited to participate (502 subjects). Five subjects were excluded due to not having the required diagnosis. Other reasons for exclusion were not living independent (n = 1), not understanding Swedish (n = 1), having another neurological disease (n = 1), and not being able to answer the survey (n = 1). The final sample consisted of 287 subjects with MS (response rate 58.2%). In total there were 84 men (29.3%) and 203 women (70.7%), giving a female-to-male ratio of 2.42:1. The female-to-male ratio for the 206 subjects who did not respond to the invitation to participate in the survey was 2.38:1. Non-respondents were slightly younger than respondents, see Table 1.

**Table 1:** Differences between respondents and non-respondents, number (%), and mean ± SD (range)

	**Respondents**	**Non-respondents**	**P-value**
**All**, n	287	206	
**Men,** n (%)	84 (29.3%)	61 (29.6%)	0.934
**Women**, n (%)	203 (70.7%)	145 (70.4%)	
**Age,** years	51.5 **±** 13.5	49.0 ± 13.4	0.048*
	(19–79)	(19–80)	
**Men,** years	52.5 ± 14.5	46.7 ± 14.0	0.018*
	(19–79)	(19–73)	
**Women,** years	51.0 ± 13.0	49.9 ± 13.0	0.463
	(22–79)	(22–80)	

*p < 0.05.

### Measurements

For all measurements the Swedish versions of the scales were used. For this study four measurements (PADS-R, ACTIVLIM, ESES, SIPA) were translated into Swedish, since no previous Swedish version existed. The translation process was following standards mainly from works by Beaton et al. [40] and Hilton and Skrutkowski [41]. For the other scales previous translations were used.

Primary outcome variables:

*Physical activity* was measured using the Physical Activity Disability Survey – Revised (PADS-R) [42]. The PADS-R was developed to measure physical activity in people with chronic illness and/or disability [43]. The survey includes six subscales; exercise, leisure time physical activity, general activity, therapy, employment and wheelchair use. For each scale the amount of physical activity during the previous week is reported. The ratings from the subscales are summed to give a total PADS-R score. The higher the score, the higher the level of physical activity. A high intra-class correlation coefficient and acceptable limits of agreement are demonstrated for test-retest reliability [42].

*Physical and psychological consequences of disease* was measured using the Multiple Sclerosis Impact Scale (MSIS-29) [44]. This scale includes 29 questions regarding the impact of MS on daily life over the previous two weeks (answers from one = not at all, to five = extremely). Twenty items concern physical consequences and nine concern psychological consequences. Summary scores for each scale are transformed to a 0–100 scale, where high values indicate more severe consequences. The scale demonstrates evidence of validity, high internal consistency, high reliability and responsiveness [44,45]. The physical scale was used in this study to classify the participants in four groups, indicating level of disease impact defined as 0–25 (minimal disease), >25-50 (mild disease), >50-75 (moderate disease) , >75-100 (severe disease) [46].

*Fatigue* was measured with the Fatigue Severity Scale (FSS) [47,48]. This scale includes nine questions to identify features of fatigue. Each question has alternative answers ranging from one (strongly disagree) to seven (strongly agree), and a total FSS is calculated by taking the mean of the nine questions. A higher score indicates a more severe level of fatigue [47]. A FSS score ≥4 can be used as a cut-off score for the presence of fatigue [49]. The English and the Swedish versions have been found to be reliable and valid [47,48].

*Self-efficacy for physical activity* was measured using the Exercise Self-Efficacy Scale (ESES) [50]. This scale includes 10 items concerning confidence when carrying out regular physical activities and exercise. Each item has four answer options (1 = not at all true, 2 = rarely true, 3 = moderately true, 4 = always true). The scores are summed to yield a total score (maximum 40 p). A high score indicates higher exercise self-efficacy. High internal consistency, good scale integrity and satisfactory content validity have been shown [50]*.*

*Fall-related self-efficacy* was measured using the Falls Efficacy Scale (Swedish version) (FES(S)) [51]. The original scale measures the degree of confidence to performing 10 common daily activities without falling [29]. In the Swedish version three additional items are included [51] The items are graded on a visual scale from not confident at all (0) to completely confident ([10]). The FES(S) is divided into two subscales 1) Personal Activities of Daily Living (PADL), items 1–6 and 2) Instrumental Activities of Daily Living (IADL) items 8–13. Item no. 7 (walking up and down stairs) is regarded as a transitory item. All items can be summed to yield a total score (maximum 130 p). A high score indicates higher self-efficacy. The use of FES(S) has shown high test-retest reliability and has also been found to be responsive to changes [52].

*Social support for physical activity* was measured using Social Influences on Physical Activity (SIPA) [53]. This is a multidimensional scale and includes social support i.e. positive social influence (SIPApos) and negative social influences (SIPAneg) [53]. SIPApos includes questions on dimensions of companionship, informational and esteem support whereas SIPAneg includes questions on inhibitive, justifying and criticizing behaviour [53]. A 5-point scale (from 0 = never to 4 = very often) is used to measure the occurrence of social influences over the last 12 months. For each item the influences of three different sources are estimated; family, friends and experts. The scale includes 27 items, where 12 items measure negative social influence and 15 measure positive social influences. For each subscale the results for family/friends/experts can be calculated as the sum of all items divided by 12 (SIPAneg) and 15 (SIPApos). A total result for the subscales can also be calculated as the sum of all items for family/friend/experts divided by 36 (SIPAneg) and 45 (SIPApos). Evidence of content validity and reliability has been shown [53,54].

Secondary and background variables were also assessed.

*Activity limitation* was measured using the ACTIVLIM questionnaire (http://www.rehab-scales.org) [55]. Activity limitations are defined as difficulties a person might have in executing activities of daily living. The part of the questionnaire for adults consists of 18 daily activities. Each question has four answer alternatives; impossible, difficult, easy and question mark (difficulty cannot be estimated since the activity has never been tried). The total score for the questionnaire is expressed in logits. A high score corresponds to a high activity level. The questionnaire exhibits very good psychometric qualities, including reliability, construct validity, reproducibility, linearity and unidimensionality [55] and has a good sensitivity to change [56].

*Gait ability* was estimated by the questions “Can you walk?” and “How far can you walk outside during the summertime?”

A yes or no question on fear of falling was also included (“Are you afraid of falling?”).

Co-morbidity was assessed with an open question regarding other health problems. From the open question, the Functional Comorbidity Index (FCI) [57] was used afterwards by the first author to count number of co-morbidities. FCI was developed with physical function as the outcome. This has shown stronger association with physical function than Charlson and Kaplan-Feinstein indices, and FCI correctly classified patients into high and low function groups in 77% of cases [57]. Body Mass Index (BMI) was calculated by dividing body weight by height squared (kg/m^2^) [58].

### Procedure

After review of the Swedish population register for the addresses of eligible participants, a questionnaire was sent out by surface mail. An informatory letter, a written consent form, and a stamped reply envelope were included with the questionnaire. The self-assessment questionnaire was divided in two parts, due to the length of the questionnaire, with the second part sent out two weeks after a reply with answers to the first part of the questionnaire was received. The first part consisted of questions about consequences of MS, activity limitation, physical activity and background questions. The second part included questions on self-efficacy and social support for physical activity, fall-related self-efficacy, fatigue and gait. Two reminders were sent to subjects who did not answer either of the parts in three weeks. All participants in the study provided written informed consent to participate. The study was approved by the Regional Ethical Review Board, Uppsala, Sweden, D-no 2010/278.

### Statistical methods

Data was analyzed using SPSS (Statistical Package for Social Sciences, version 20). Missing values for occasional data in scales with ordinal questions were imputed with median for the appropriate subscale when the level of missing data was <33% of the subscale. When the amount of missing data was ≥33% the total of the scale was not calculated. For the different measures this was the case in six MSIS-29 physical and psychological, two FSS, one ACTIVLIM and PADS-R, three ESES, four FES(S), eight SIPAneg and 13 SIPApos cases i.e. 4.5% at most. In a few cases ([3,4]) height and weight were missing. These values were imputed by the mean for men or women. Subgroup analyses were performed to explore if the gender differences were caused by the men being more severely affected by the disease. The subgroup analyses focused on physical consequences of disease (MSIS-29 physical scale) and type of MS. After checks of normal distribution differences between men and women were analyzed with Chi-square test, Mann–Whitney U-test or Student’s t-test depending on the type of scale and normal distribution. The level of significance was set at p < 0.05.

## Results

Background characteristics and secondary outcome measures are presented in Table 2. The mean age of the study cohort was 51.5 ± 13.5 years, and the median time since diagnosis was 11 years. Type of MS was relapsing remitting or secondary progressive in 83% of the subjects. No differences between the genders were found for most of the background variables except that the men had been diagnosed for a significantly longer time. Men were more limited in their activity and walked shorter distances outdoors (Table 2). In addition, 20.2% men versus 6.4% women were not able to walk.

**Table 2:** Background characteristics, and secondary outcome measures, mean ± SD (range), median (IQ-range), or number (%)

		**All**	**Men**	**Women**	**P-value**
**Age** (years)		51.5 ± 13.5 (19–79)	52.5 ± 14.5 (19–79)	51.0 ± 13.0 (22–79)	0.397
**Type of MS**	relapsing remitting	135 (47.0%)	26 (31.0%)	109 (53.7%)	0.450
	secondary progressive	104 (36.2%)	37 (44.0%)	67 (33.0%)	
	primary progressive	32 (11.1%)	16 (19.0%)	16 (7.9%)	
	progressive relapsing	10 (3.5%)	4 (4.8%)	6 (3.0%)	
	subtype not known	6 (2.1%)	1 (1.2%)	5 (2.5%)	
**Time since diagnosis** (years)		11 (11–18)	12 (7–22)	10 (5–18)	0.027*
**Living with partner**	alone	80 (27.9%)	27 (32.1%)	53 (26.1%)	0.266
	with partner	203 (70.7%)	55 (65.5%)	148 (72.9%)	
**Living with children**	without children	216 (75.3%)	66 (78.6%)	150 (73.9%)	0.403
	with children	71 (24.7%)	18 (21.4%)	53 (26.1%)	
**Education**	compulsory school	39 (13.6%)	14 (16.7%)	25 (12.3%)	0.473
	upper secondary school	118 (41.1%)	34 (40.5%)	84 (41.1%)	
	post-secondary school	124 (43.2%)	32 (38.1%)	92 (45.3%)	
**Work status**	working	118 (41.1%)	29 (34.5%)	89 (43.8%)	0.398
	student	4 (1.4%)	1 (1.2%)	3 (1.5%)	
	out of work	8 (2.8%)	2 (2.4%)	6 (3.0%)	
	parental leave	4 (1.4%)	1 (1.2%)	3 (1.5%)	
	sickness benefit	52 (18.1%)	16 (19.0%)	36 (17.7%)	
	retired due to age	61 (21.3%)	24 (28.6%)	37 (18.2%)	
	parttime work/sickness benefit	12 (4.2%)	1 (1.2%)	11 (5.4%)	
	other	23 (8.0%)	8 (9.5%)	15 (7.4%)	
**Height** (cm)		170.4 ± 8.1	179.0 ± 5.6	166.8 ± 5.9	N.A.
**Weight** (kg)		71.8 ± 12.2	79.4 ± 11.0	68.7 ± 11.3	N.A.
**BMI** (kg/m^2^)		24.7 ± 3.8	24.7 ± 3.0	24.7 ± 4.1	0.926
**Smoking**	yes	41 (14.3%)	12 (14.3%)	29 (14.3%)	0.952
	no	243 (84.7%)	70 (83.3%)	173 (85.2%)	
**Other diseases**	0	212 (73.9%)	67 (79.8%)	145 (71.4%)	0.152
	1	58 (20.2%)	13 (15.5%)	45 (22.2%)	
	2	14 (4.9%)	3 (3.6%)	11 (5.4%)	
	3	3 (1.0%)	1 (1.2%)	2 (1.0%)	
**ACTIVLIM**		3.2 (1.1–6.3)	1.8 (-0.2–6.2)	3.7 (1.5–6.3)	0.002*
**Gait Y/N**	yes can walk	256 (89.2%)	67 (79.8%)	189 (93.1%)	0.001*
	no can’t walk	30 (10.5%)	17 (20.2%)	13 (6.4%)	
**Gait outside†**	1–50 m	15 (10.5%)	5 (6.0%)	10 (4.9%)	0.001*
	51–100 m	19 (5.2%)	8 (9.5%)	11 (5.4%)	
	101–500 m	30 (10.5%)	9 (10.7%)	21 (10.3%)	
	501–1000 m	5 (8.7%)	4 (4.8%)	21 (10.3%)	
	1001–3000 m	33 (11.5%)	9 (10.7%)	24 (11.8%)	
	>3000 m	110 (38.3%)	25 (29.8%)	85 (41.9%)	
**Fear of falling**	yes	109 (38%)	32 (38.1%)	77 (37.9%)	0.951
	no	162 (56.4%)	47 (56.0%)	115 (56.7%)	

*p < 0.05, N.A. = not applicable. † = total sum not 100% because those who could not walk are not displayed.

The primary outcome measures are presented in Table 3. The mean physical activity level was 0.184 ± 1.475. Physical activity levels were lower in men, and 65.5% of the men versus 47.8% of the women had an activity level less than the mean value. The median physical and psychological consequences of the disease (MSIS-29) were 27.5 and 27.8 respectively. Men were more physically affected than women with a median physical value of 41.3 versus 25.0 for women, p = 0.004. Severe disease was found in 17.9% of the men and 5.4% of the women. Fatigue and psychological consequences of the disease were similar between the genders. The medians for self-efficacy for physical activity and fall-related self-efficacy were 27 and 112 respectively for the whole sample with significant gender differences. Men had significantly lower physical activity self-efficacy (ESES) and fall-related self-efficacy (FES(S)) compared to women. Social support for physical activity was similar between the genders, despite the finding that men received less positive support from family compared to women.

**Table 3:** Primary outcome measures, mean ± SD, median (IQ-range), or number (%)

		**All**	**Men**	**Women**	**P-value**
**PADS-R**	total	0.184 ± 1.475	–0.180 ± 1.632	0.333 ± 1.383	0.007*
	≥ mean	135 (47.0%)	29 (34.5%)	106 (52.2%)	0.006*
	< mean	152 (53.0%)	55 (65.5%)	97 (47.8%)	
**MSIS-29**	physical	27.5 (7.5–52.5)	41.3 (13.8–63.8)	25.0 (6.3–47.5)	0.004*
	psychological	27.8 (11.1–47.2)	30.6 (13.9–50.0)	25.0 (9.0–43.8)	0.18
	physical 0–25	133 (46.3%)	31 (36.9%)	102 (50.2%)	0.002*
	physical >25–50	71 (24.7%)	15 (17.9%)	56 (27.6%)	
	physical >50–75	51 (17.8%)	20 (23.8%)	31 (15.3%)	
	physical >75–100	26 (9.1%)	15 (17.9%)	11 (5.4%)	
**FSS**	total	4.66 (2.94–5.89)	4.78 (3.11–6.11)	4.56 (2.9–5.89)	0.417
	≥4	177 (61.7%)	54 (64.3%)	123 (60.6%)	0.51
	<4	108 (37.6%)	29 (34.5%)	79 (38.9%)	
**ESES**		27 (20–35)	25 (18–33)	28 (21–36)	0.013*
**FES(S)**	total	112 (65–129)	99 (40–128)	116 (76–130)	0.012*
	IADL	52 (26–60)	41 (12–60)	54 (29–60)	0.016*
	PADL	54 (36–60)	51 (25–59)	56 (42–60)	0.003*
**SIPAneg**	total	0.06 (0.00–0.28)	0.08 (0.00–0.33)	0.06 (0.00–0.25)	0.067
	family	0.08 (0.00–0.33)	0.13 (0.00–0.58)	0.08 (0.00–0.33)	0.558
	friends	0.00 (0.00–0.33)	0.08 (0.00–0.33)	0.00 (0.00–0.33)	0.454
	experts	0.00 (0.00–0.08)	0.00 (0.00–0.17)	0.00 (0.00–0.08)	0.215
**SIPApos**	total	0.80 (0.40–1.36)	0.73 (0.36–1.25)	0.82 (0.40–1.38)	0.382
	family	1.13 (0.45–1.75)	1.00 (0.55–1.70)	1.23 (0.40–1.80)	0.005*
	friends	0.67 (0.20–1.40)	0.60 (0.08–1.07)	0.80 (0.27–1.47)	0.139
	experts	0.23 (0.00–1.15)	0.40 (0.00–1.25)	0.20 (0.00–1.07)	0.38

*p < 0.05, MSIS-29 = Multiple Sclerosis Impact Scale, FSS = Fatigue Severity Scale, PADS-R = Physical Activity Disability Survey – Revised, ESES = SCI Exercise Self-Efficacy Scale, FES(S) = Falls Efficacy Scale Swedish version, IADL = Instrumental Activities of Daily Living, PADL = Personal Activities of Daily Living, SIPAneg = Social Influences on Physical Activity negative subscale, SIPApos = Social Influences on Physical Activity positive subscale.

Physical consequences of disease subgroup analyses (MSIS-29 physical scale) showed that the differences between the genders found in the whole sample disappeared. But another difference emerged; women with moderate MS experienced higher level of fatigue than men (Table 4).

**Table 4:** Physical consequences of disease subgroup analysis (MSIS-29 physical scale), mean ± SD, median (IQ-range)

**MSIS-29 0–25, minimal MS**				
		**Men (n = 31)**	**Women (n = 102)**	**P-value**
**PADS-R**	total	1.213 ± 1.389	1.094 ± 1.063	0.613
**FSS**	total	3.44 (1.78–5.11)	3.39 (2.22–4.58)	0.831
**ESES**		32 (25–35)	35 (27–40)	0.103
**FES(S)**	total	129 (123–130)	129 (122–130)	0.998
**SIPApos**	family	0.90 (0.33–1.65)	1.27 (0.40–1.82)	0.263
**MSIS-29 ≤ 50, minimal and mild MS**				
		**Men (n = 46)**	**Women (n = 158)**	**P-value**
**PADS-R**	total	0.807 ± 1.442	0.727 ± 1.188	0.704
**FSS**	total	4.00 (2.33–5.36)	4.28 (2.56–5.22)	0.595
**ESES**		29 (23–35)	30 (23–37)	0.388
**FES(S)**	total	124 (100–130)	125 (103–130)	0.941
**SIPApos**	family	1.00 (0.60–1.57)	1.27 (0.40–1.80)	0.603
**MSIS-29 > 50**–**75, moderate MS**				
		**Men (n = 20)**	**Women (n = 31)**	**P-value**
**PADS-R**	total	-1.031 ± 1.006	-0.843 ± 0.892	0.486
**FSS**	total	5.89 (4.25–6.42)	6.50 (5.57–6.89)	0.047*
**ESES**		20 (16–29)	22 (16–28)	1.000
**FES(S)**	total	63 (34–87)	48 (29–81)	0.387
**SIPApos**	family	0.93 (0.20–1.47)	1.33 (0.72–1.73)	0.150
**MSIS-29 > 50–100, moderate and severe MS**				
		**Men (n = 35)**	**Women (n = 42)**	**P-value**
**PADS-R**	total	-1.332 ± 0.933	-1.147 ± 1.031	0.415
**FSS**	total	6.00 (4.50–6.58)	6.44 (5.50–6.89)	0.193
**ESES**		18 (13–26)	20 (15–26)	0.542
**FES(S)**	total	41 (10–69)	40 (21–60)	0.935
**SIPApos**	family	1.00 (0.28–1.83)	1.27 (0.57–1.73)	0.528

* p < 0.05, MSIS29 = Multiple Sclerosis Impact Scale, PADS-R = Physical Activity Disability Survey – Revised, FSS = Fatigue Severity Scale ESES = SCI Exercise Self-Efficacy Scale, FES(S) = Falls Efficacy Scale Swedish version, SIPApos = Social Influences on Physical Activity positive subscale.

Women with relapsing remitting MS experienced higher level of fatigue than men. Men with secondary progressive MS were less physically active than the women, which might be explained by the more severe physical consequences of the disease (Table 5).

**Table 5:** Diagnosis subtype subgroup analysis, mean ± SD, median (IQ-range)

**MS type, relapsing remitting**				
		**Men (n = 26)**	**Women (n = 109)**	**P-value**
**MSIS-29**	physical	8.8 (1.3–23.8)	11.3 (2.5–30.0)	0.235
	psychological	16.7 (8.3–38.9)	25.0 (8.3–41.7)	0.336
**PADS-R**	total	0.933 ± 1.612	0.840 ± 1.240	0.745
**FSS**	total	3.22 (1.78–5.14)	4.33 (2.89–5.67)	0.048*
**ESES**		29 (22–39)	32 (26–37)	0.571
**FES(S)**	total	129 (111–130)	128 (111–130)	0.927
**SIPApos**	family	0.97 (0.60–1.55)	1.37 (0.57–1.87)	0.280
**MS type, secondary progressive**				
		**Men (n = 37)**	**Women (n = 67)**	**P-value**
**MSIS-29**	physical	53.8 (28.8–77.5)	40.0 (23.8–58.8)	0.019*
	psychological	33.3 (16.7–47.2)	25.0 (11.1–41.7)	0.346
**PADS-R**	total	-0.874 ± 1.266	-0.349 ± 1.241	0.043*
**FSS**	total	5.89 (3.78–6.39)	5.22 (3.44–6.44)	0.390
**ESES**		24 (18–33)	24 (16–32)	0.981
**FES(S)**	total	66 (31–103)	82 (48–115)	0.289
**SIPApos**	family	0.93 (0.30–1.87)	1.13 (0.27–1.70)	0.784

* p < 0.05, MSIS-29 = Multiple Sclerosis Impact Scale, PADS-R = Physical Activity Disability Survey – Revised, FSS = Fatigue Severity Scale ESES = SCI Exercise Self-Efficacy Scale, FES(S) = Falls Efficacy Scale Swedish version, SIPApos = Social Influences on Physical Activity positive subscale.

## Discussion

Our results showed, in a Swedish sample of subjects with MS, that men were less physically active, had lower levels of self-efficacy for physical activity and fall-related self-efficacy, and received less social support from family. These gender differences were explained by men being more physically affected by the disease. The psychological consequences of the disease were similar between the genders. Subgroup analysis showed that women with moderate physical consequences of the disease and women with relapsing remitting MS experienced more fatigue than men.

### Physical activity

Men were found to be less physically active, both when comparing the mean PADS-R value and when splitting the sample in two groups (≥ mean/< mean). In accordance with our results a meta-analysis by Motl et al. [8] has shown combined samples of women and men to be significantly less physically active than samples solely of women. The lower activity level in men was in our study explained by them being more physically affected by the disease. Thus, the genders were equally active when consideration was taken to physical consequences of the disease. Our results are in accordance with other studies reporting that MS subjects with more severe disease are less physically active [11,23].

The fact that men and women with MS were equally active is in contrast to results from the general population, where men are more physically active [35]. One explanation might be a gender bias in the measurements used, i.e. underestimation of physical activity in women, as many studies have focused on activities associated with sports and exercise (traditionally performed by men) and less on activities performed by women, such as housework, gardening and walking [59]. However, the PADS-R used in this study measures a wide range of activities that may better reflect the overall physical activity levels in both men and women [60]. All physical activity is of benefit, but WHO recommends at least 150 min of moderate-intensity aerobic physical activity throughout the week, but for additional health benefits this activity should be increased to at least 300 min [14]. However, it is not known yet which value on the PADS-R that equivalents this level of physical activity. The mean physical activity level measured with PADS-R was 0.184 which is slightly higher than in another MS sample (n = 287) where the mean PADS-R score was 0.02 ± 1.32 [25].

### Severity of disease and fatigue

In this study a large gender difference was found in the severity of disease as measured with MSIS-29. Men were also more limited in their activity and walking ability than women. Although men experienced significantly more severe physical consequences of the disease there were no differences between the genders in psychological consequences. Subgroup analyses showed that men with secondary progressive MS were much more physically affected by the disease, but for relapsing remitting MS no significant differences in consequences of the disease, as measured with MSIS-29, were found. This is in accordance with other studies summarized by Jobin et al. [39] showing that a benign course of the disease is more often associated with female gender, and the progression is worse for men compared to women.

There was no gender difference in fatigue in the whole sample, but women with relapsing remitting MS, and moderate MS experienced more fatigue than men (Tables 4 and 5). Previous reports on fatigue in MS are inconclusive [39,61,62].

### Self-efficacy for physical activity

Self-efficacy for physical activity was high, which in accordance with another study in subjects with MS [23]. Men in our study rated self-efficacy for physical activity lower than women, but this difference was not maintained when physical consequences of disease and disease subtypes were included in the analyses. Thus, the lower self-efficacy for physical activity in men may be explained by the more physically affected and less physically active men. Social support enhances a person’s level of self-efficacy [27]. Women in our study had significantly more social support for physical activity from family members, which might have contributed to the higher level of self-efficacy in women.

### Fall-related self-efficacy

We found that fall-related self-efficacy was high in the total sample, but lower in men compared to women. Again the gender difference in our data was explained by men being more affected by the disease. Our data is in accordance with another study reporting that experiencing more MS symptom interference during everyday activities is associated with fear of falling [31]. The FES(S) has to our knowledge not been previously used to examine subjects with MS. The English version has been used for persons with MS and correlated with balance parameters and improved after progressive resistance training [18]. The English and Swedish versions are not equivalent due to the different number and ordering of activities.

### Social support for physical activity

The most important source of positive influence in our study and also in previous reports [53,54] was found to be from family. Women in this sample received more social support from family than men, but no other gender differences were found for all other positive or negatives influences. However, both subgroups analyses showed no significant gender differences. A reason for this could be that the gender difference was rather small, and only statistically significant in the total sample. SIPA was chosen to measure social support for physical activity because it measures both social support and negative social influences that are considered to be different constructs [54]. In this study the negative influences were low, which is positive. Some negative influences were projected to persons with MS from family members, but were nearly absent from friends and experts. In this study a large floor effect was found for the negative social influence subscale, with a mean of zero for 36.2% of the total and even higher values for subscales (family 46.0%, friends 51.2%, and experts 69.0%). Due to these findings the future use of this subscale for persons with MS in Sweden cannot be recommended, but a floor effect for SIPApos was only found for the expert subscale (34.8%).

### Generalization and limitations

The Swedish Multiple Sclerosis Registry, from where our subjects were recruited, is a register where patients with MS or possible MS have been entered [2], which implies a risk for inclusion of subjects not actually having the diagnosis. We found five subjects who did not have the required diagnosis for inclusion.

In our study the mean age was 51.5 years and the female-to-male ratio 2.42:1. This is close to the nationwide Swedish MS study (n = 17 485), where the mean age was 52.6 years and the female-to-male ratio 2.35:1 [2], which strengthen the external validity of our results and extrapolation to other individuals with MS in Sweden.

It is important to keep in mind when generalizing our result that this study sample consisted of persons living at home with relatively low consequences of MS. The consequences of disease in our sample were lower than in two other MS samples where the mean MSIS-29 was 45.5-57.2 on the physical scale and 46.2-67.2 on the psychological scale [45,46]. One reason for the more affected samples in other studies could be the focus on subjects with more severe MS and more clinical contact [45,46].

The response rate in this sample cohort was 58.2%. This is similar to the mean response rate of 60% for published studies in medical journals [63] and for other MS studies using mailed surveys (response rates 31-77% [6,23,25,44,46,61,64]). The response rate itself is not, however, a sufficient measure of the quality of a study if there are enough cases for statistical analyses, although there are more possibilities of non-response bias [63]. In this study there were enough respondents for statistical analyses, but a non-response bias was also found. The non-respondents were younger. One reason for this could be that younger subjects experienced fewer symptoms from the disease and considered the questions not suitable, or perhaps they were even emotionally stressed by questions about subjects such as symptoms and gait ability.

Poor design of a questionnaire will result in a failure to measure what is required [65]. In this study measurements with adequate reliability and validity were used. However, there are some limitations. Four of the measurements have recently been translated from English into Swedish, and only two of the Swedish translations have yet been psychometrically tested. ESES showed satisfying results but there were not enough subjects to draw conclusions from SIPA (unpublished results). A strength is however that our research group did make efforts to perform a thorough process of translation. Ceiling effects were found for ACTIVLIM (31.5%) and FES(S) (23.3%) that have not yet been psychometrically evaluated in individuals with MS. The total questionnaire included many questions. After pilot-reading of the questionnaire by representatives from a patient organization, Neuro Sweden, it was decided in discussions with the representatives to separate the survey into two parts.

### Clinical implications

Physical activity behavior to promote health and minimize secondary complications of inactivity such as coronary heart disease and type II diabetes [66] should be emphasized in patients with MS. Persons with low levels of physical activity need more support from health professionals to increase physical activity behavior. Our results show that this particularly concerns men, and it can be explained by them being more physically affected by the disease. Probably individualized and adapted physical activity support is of value. It is important for clinicians to use the family as a resource in patient-rehabilitation. Some women experience more fatigue than men, and might need more support from clinicians to overcome this barrier to physical activity. Our results are of value in achieving a better understanding of gender differences.

## Conclusions

This cohort study of physical activity levels in subjects with MS in Sweden demonstrated that men were less physically active, and had lower levels of self-efficacy for physical activity and fall-related self-efficacy. These gender differences were explained by men being more physically affected by the disease. The study also identified that men received less social support from family members. Subgroups of women with moderate MS and relapsing remitting MS experienced more fatigue than men. Few studies have been focusing on gender and physical activity, and future studies are of value.

## Abbreviations

BMI: Body mass index; ESES: Exercise self-efficacy Scale; FES(S): Falls efficacy scale (Swedish version); FSS: Fatigue Severity Scale; MS: Multiple sclerosis; MSIS-29: Multiple sclerosis impact scale; PADS-R: Physical activity disability survey – revised; SIPA: Social influences on physical activity; SIPAneg: Social influences on physical activity - negative social influences; SIPApos: Social influences on physical activity - positive social influences.

## Competing interests

The authors declare that they have no competing interests.

## Authors’ contributions

The initiative and planning of the study and translation of questionnaires was a joint effort between EA, ME and KH. EA was most responsible for the progression of the study, data analysis, interpretation of data and drafting the manuscript. ME also contributed to the interpretation of data and revision of the manuscript. LZ contributed in data analysis and revision of the manuscript. KH also contributed to data analysis, interpretation of data and revision of the manuscript. All authors read and approved the final manuscript.

## Pre-publication history

The pre-publication history for this paper can be accessed here:

http://www.biomedcentral.com/1471-2377/14/47/prepub
